# Posttranslational Modifications of the Mineralocorticoid Receptor and Cardiovascular Aging

**DOI:** 10.3389/fmolb.2021.667990

**Published:** 2021-05-28

**Authors:** Yekatarina Gadasheva, Alexander Nolze, Claudia Grossmann

**Affiliations:** Julius-Bernstein-Institute of Physiology, Martin Luther University Halle-Wittenberg, Halle (Saale), Germany

**Keywords:** mineralocorticoid receptor, cardiovascular aging, posttranslational modification, phosphorylation, acetylation, ubiquitination, sumoylation, oxidation

## Abstract

During aging, the cardiovascular system is especially prone to a decline in function and to life-expectancy limiting diseases. Cardiovascular aging is associated with increased arterial stiffness and vasoconstriction as well as left ventricular hypertrophy and reduced diastolic function. Pathological changes include endothelial dysfunction, atherosclerosis, fibrosis, hypertrophy, inflammation, and changes in micromilieu with increased production of reactive oxygen and nitrogen species. The renin-angiotensin-aldosterone-system is an important mediator of electrolyte and blood pressure homeostasis and a key contributor to pathological remodeling processes of the cardiovascular system. Its effects are partially conveyed by the mineralocorticoid receptor (MR), a ligand-dependent transcription factor, whose activity increases during aging and cardiovascular diseases without correlating changes of its ligand aldosterone. There is growing evidence that the MR can be enzymatically and non-enzymatically modified and that these modifications contribute to ligand-independent modulation of MR activity. Modifications reported so far include phosphorylation, acetylation, ubiquitination, sumoylation and changes induced by nitrosative and oxidative stress. This review focuses on the different posttranslational modifications of the MR, their impact on MR function and degradation and the possible implications for cardiovascular aging and diseases.

## Introduction

### Mineralocorticoid Receptor Gene and Protein Structure

The MR is a ligand-dependent transcription factor with aldosterone as an endogenous ligand in humans. Cloning of the MR occurred relatively late compared to the other steroid receptors (Arriza et al., 1987; Morrison et al., 1990). The human MR gene comprises ten exons, of which eight are translated (Zennaro et al., 1995). Exon 1a and 1b act as functional promoters, and alternative transcription of the two 5′-UTRs generates the isoforms hMRa and hMRb, which are co-expressed in MR target tissues (Zennaro et al., 1997). The translational start site of the MR is located within exon 2 so that both isoforms lead to the same 107 kDa hMR protein. Exon 2 gives rise to the N-terminal domain (NTD), which is the longest and most variable domain compared to other steroid hormone receptors. Exon 3 and 4 each encode one of the two zinc fingers of the DNA-binding domain (DBD) while the last five exons encode the ligand-binding domain of the MR (LBD).

**FIGURE 1:**
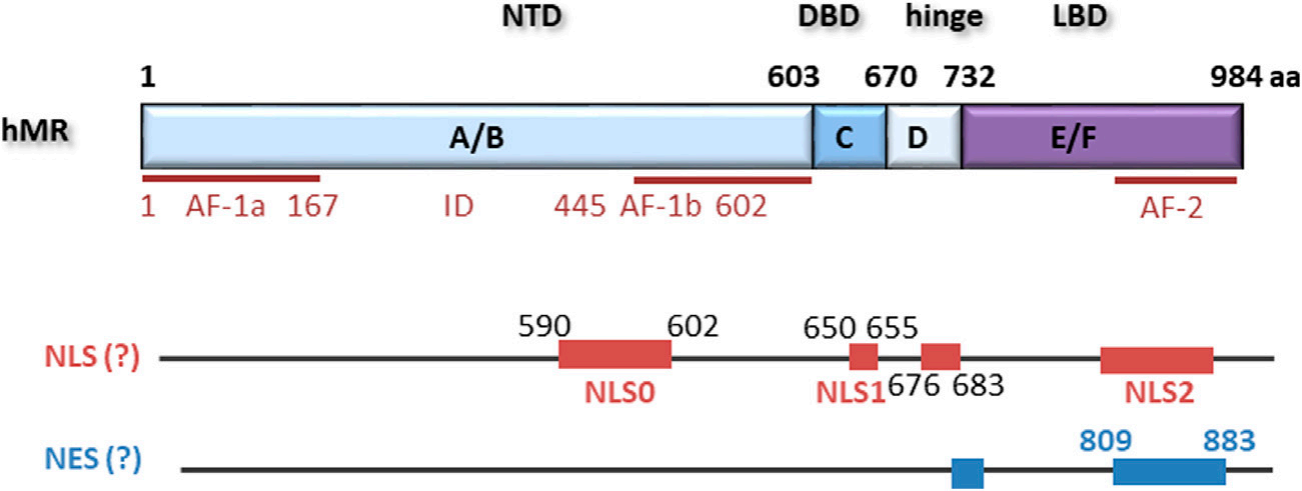
hMR comprises 984 amino acids with an N-terminal domain (NTD), a DNA-binding domain (DBD), a hinge region and a ligand binding domain (LBD). Located within the NTD is an activation function 1 (AF-1) that is divided by an inhibitory domain (ID) into two parts, AF-1a and AF-1b. AF-2 is located in the LBD. Several nuclear localization signals (NLS) and putative nuclear export signals (NES) have been described.

The N-terminal A/B domain is highly evolutionarily conserved but possesses less than 15% homology with the other steroid receptors. It contains an autonomous activation functional domain (AF-1) consisting of two parts, AF-1a and AF-1b, separated by an inhibitory domain (ID) (Figure 1). AF-1 is constitutively active but suppressed by the C-terminal E/F domain in the absence of ligand. Furthermore, the NTD is responsible for modulating selectivity of the MR for ligand and cofactor interactions (Jausonsloffreda et al., 1994). It also contains a serine/threonine-rich nuclear localization signal NLS0 involved in ligand-independent nuclear localization of MR (Walther et al., 2005). Adjacent to the A/B domain is the C domain containing the DBD. The DBD is highly conserved among steroid receptors, contains two zinc fingers, and binds to GRE hormone response elements of the DNA. The N-terminal zinc finger is responsible for the protein-DNA interaction and contains a P box that leads to interaction with a half-sites of a GRE by intercalating into the major groove. The second zinc finger contains a D box and is responsible for weak MR dimerization (Sartorato et al., 2004). A bipartite basic motif-containing nuclear localization site 1 (NLS1) is localized at the C terminus of the DBD and stretches into the hinge region. The short hinge region connects the DBD and the LBD and contains multiple prolines, which lead to a twist that allows the receptor to contact the transcriptional machinery (Tsai and O'Malley, 1994). The LBD of the MR consists of twelve α-helices and one beta-sheet formation in three antiparallel layers (Hellal-Levy et al., 2000; Farman and Rafestin-Oblin, 2001). In its unliganded state, the MR is bound to chaperone proteins, including heat shock proteins and immunophilins. Besides a ligand-binding pocket, the LBD also possesses several chaperone interaction points, a nuclear localization signal, an area involved in heterodimerization with the GR, and the transcriptional activation functional domain 2 (AF-2) (Savory et al., 2001; Bain et al., 2007). Binding of aldosterone causes conformational changes, which lead to activation of AF-2 and released suppression of the AF-1 function in the NTD (Hong et al., 1997; Fuse et al., 2000). Although AF-1 and AF-2 can act independently, both are required for full ligand-dependent MR transactivation. Additionally, cofactor binding sites are exposed, leading to the recruitment of coactivators, for example members of the steroid receptor coactivator family or GRIP1 (Bain et al., 2007; Hong et al., 1997; Tsai and O'Malley, 1994). The conformational change also was reported to unmask the nuclear localization signal 2 (NLS2) (Sanchez et al., 1985; Walther et al., 2005; Galigniana et al., 2010).

### Mineralocorticoid Receptor Signaling

The MR and its closest relative the glucocorticoid receptor (GR) evolved from a common corticoid receptor. Evolutionwise, a separation into MR and GR was first found in cartilaginous fish. However, these fish do not possess distinct ligands for the two receptors, so that 11-desoxycorticosterone, glucocorticoids but also progesterone act as natural ligands for both receptors (Sturm et al., 2005; Baker and Katsu, 2017). In lungfish, aldosterone occurs for the first time, and in terrestrial vertebrates, aldosterone typically is the specific ligand for the MR (Joss et al., 1994; Rossier et al., 2015). In humans, the enzyme 11β hydroxysteroid dehydrogenase 2 (11β -HSD2) is located near MR and inactivates glucocorticoids to prevent them from accessing the MR. In tissues with low 11β-HSD2 activity, glucocorticoids bind to MR with comparable affinity to aldosterone.

In the absence of ligand, the MR is primarily located in the cytosol (Nishi et al., 2004; Piwien Pilipuk et al., 2007), or it is equally distributed between cytosol and nucleus depending on cell type and cellular context (Fejes-Toth et al., 1998; Nishi et al., 2001; Tanaka et al., 2005; Walther et al., 2005). In cells with low HSP90 content like cardiomyocytes, it has been demonstrated to be constitutively in the nucleus (Hernandez-Diaz et al., 2010). Nevertheless, MR without ligand is transcriptionally inert. In the cytosol, the MR is associated with chaperones including HSP90, HSP70, and p23 and various immunophilins like FKBP51 (Sanchez et al., 1985; Galigniana et al., 2010) that prevent degradation and ensures a conformation that allows binding of ligand (Caamano et al., 1993; Binart et al., 1995). Upon binding of ligand, a change in associated proteins occurs, and the MR rapidly translocates into the nucleus, where it becomes transcriptionally active (Piwien Pilipuk et al., 2007; Grossmann et al., 2012). Nuclear import of MR is controlled through three nuclear localization signals (Walther et al., 2005). Nuclear localization of naive MR is mediated primarily through NLS0 in the NTD. Specific amino acid substitutions that mimicked phosphorylation selectively enhanced or repressed NLS0 activity suggesting potential regulation sites (Walther et al., 2005). Nuclear transfer through NLS2 within the LBD is dependent on steroid agonists. The third NLS (NLS1) acts together with NLS0 and NLS2 to stimulate nuclear uptake of the agonist-treated receptor, but also directs the complete nuclear localization of MR in response to treatment with steroid antagonist. Tanaka et al. demonstrate that intact NLS1 is necessary for the trafficking of MR together with importin alpha to the nucleus in COS1 cells (Tanaka et al., 2005). However, inhibition of HSP90 strongly delayed nuclear translocation without preventing complete nuclear accumulation of MR. Blocking of NLS1 also did not fully inhibit MR nuclear translocation. Therefore, HSP90- and NLS1-dependent and independent nuclear translocation mechanisms seem to exist (Walther et al., 2005; Piwien Pilipuk et al., 2007; Galigniana et al., 2010). Previously, it was hypothesized that binding of ligand causes dissociation of MR from HSP90 and thereby unmasking of NLS with associated nuclear translocation (Picard and Yamamoto, 1987; Walther et al., 2005). This finding was challenged by experiments showing that mutants without NLS but associated with NLS-containing-HSP90 also are transported into the nucleus (Kang et al., 1994). Overall, the conformational change after ligand binding seems to initiate a shift in the composition of attached chaperone molecules, allowing FKBP52 to replace FKBP51. FKBP52 then links the MR complex to dynein/dynactin motor proteins which propagate nuclear translocation (Banerjee et al., 2008; Galigniana et al., 2010). Simultaneously, several investigators found that HSP90 is not exclusively located in the cytoplasm but also in the nucleus (Galigniana et al., 2010; Hernandez-Diaz et al., 2010; Grossmann et al., 2012) and that HSP90 inhibitors can affect nuclear translocation and transcriptional activity. Additionally, HSP90 enhances DNA binding of MR without being bound to DNA itself, probably by stabilization of receptor structure (Bain et al., 2007). No transactivation of GR or MR occurs without ligand or after incubation with an HSP90 inhibitor, but the exact function of HSP90 is still under debate (Nemoto et al., 1993; Grossmann et al., 2012).

In the nucleus, the MR binds to hormone response elements on the DNA as a homodimer (Tsai et al., 1988; Wrange et al., 1989; Drouin et al., 1992) but may also be able to form heterodimers with the GR, although the relevance of these still is not clear (Liu et al., 1995; Savory et al., 2001; Nishi et al., 2004). Homodimerization of MR and other steroid receptors seems to occur as soon as MR is released from HSP90 and therefore at least *in vitro* independently of DNA or ligand (Savory et al., 2001; Grossmann et al., 2012). There are several indications that dimerization occurs in the nucleus before DNA binding. Binding of steroid receptors to their hormone response elements involves rapid cycling with the rate of exchange influencing the transcriptional activity. HSP90 has been shown to modulate receptor cycling at DNA and possibly also receptor degradation (Kang et al., 1999; Freeman and Yamamoto, 2002; Stavreva et al., 2004). However, only homodimers formed in the nucleus seem to be able to regulate gene expression.

Nuclear export, on the other hand, has not been well characterized for steroid receptors in general. For the androgen receptor, an untypical, not leucine-rich nuclear export signal was found and characterized in the LBD, which functioned independently of CRM1/exportin. Corresponding structural elements were also found in the MR, but the sequence homology is only moderate (Saporita et al., 2003). For the GR, also a CRM1-independent nuclear export that takes several hours after steroid withdrawal was described. It relies on 15 amino acids of the DBD between the two zinc fingers (Madan and DeFranco, 1993; Black et al., 2001; Walther et al., 2003). However, for the MR no export was detected, and it has been suggested that it is an atypical nuclear hormone receptor that moves unidirectionally from the cytoplasm to the nucleus (Walther et al., 2005). Of all the steroid receptors, MR signaling is the least well explored, and it is not clear how MR inactivation and degradation occurs.

## Cardiovascular Effects of Mineralocorticoid Receptor and Aging

The MR is part of the renin-angiotensin-aldosterone-system (RAAS), and angiotensin II stimulates aldosterone secretion. Classical epithelial MR effects occur in kidney, colon and sweat glands and include sodium and water reabsorption as well as proton and potassium secretion. MR effects in the kidney regulate overall volume and electrolyte homeostasis and thereby control systemic blood pressure. The MR is also expressed in various non-epithelial tissues. For example, in the vascular wall, the MR was detected in vascular smooth muscle cells (VSMCs), in endothelial cells but also fibroblasts and immune cells.

MR antagonists have proven beneficial for different cardiovascular diseases going clearly beyond a lowering of blood pressure and optimization of water-electrolyte-homeostasis. Several large clinical trials indicate that MR antagonists effectively treat heart failure with reduced ejection fraction, a typical cardiovascular disease of the elderly (Zannad et al., 2010; Pitt, 2012; Pitt et al., 2014). Overall, a reduction in mortality and morbidity was registered. Additional clinical benefits of MR antagonists were reported in patients with left ventricular hypertrophy and hypertension (Pitt et al., 2003) and in patients with atrial fibrillation (Dabrowski et al., 2010; Fudim et al., 2018). For patients with heart failure with preserved ejection fraction, the picture is less clear with no significant reduction in the primary composite outcome of death due to cardiovascular causes or hospitalization (Pitt et al., 2014). However, when looking at subgroups, some benefits of MR antagonists were found depending on the geographical origin of the patients, sex and age (Pitt et al., 2014; Pfeffer et al., 2015; Merrill et al., 2019). Recently published results show that new MR antagonists reduce the risk of chronic kidney disease progression and cardiovascular events in patients with chronic kidney disease and type 2 diabetes regardless of pre-existing cardiovascular problems (Bakris et al., 2020; Filippatos et al., 2021). Therefore, results from clinical trials suggest that an overactive RAAS is accountable for some of the cardiovascular changes occurring in cardiovascular diseases and in an attenuated form also during healthy aging.

During aging, plasma renin activity and aldosterone levels decrease, so it is not clear at first glance why RAAS should contribute to cardiovascular changes during aging (Weidmann et al., 1975; Noth et al., 1977; Tzunoda et al., 1986; Bauer, 1993). However, 11β-HSD2 activity required for preventing glucocorticoids from occupying the MR also declines with age, so that binding of cortisol to MR increases (Henschkowski et al., 2008; Campino et al., 2013). Under normal circumstances in non-epithelial tissues with low 11β-HSD2 expression, this does not necessarily activate the MR. This may change in the presence of a permissive micro-milieu found in older patients where oxidative stress and pro-inflammation as well as hypoxia may be more prevalent. Consequently, posttranslational modification of MR or associated factors may occur and affect MR activity. Nanba et al. report a decrease in physiological aldosterone secretion but an increase in autonomous aldosterone production in aged individuals, which might also explain how the RAAS contributes to cardiovascular diseases during aging (Nagarajan et al., 2017). Furthermore, the expression of MR in the vascular wall is increased during aging (Krug et al., 2010; DuPont et al., 2016). MR activation was shown to enhance angiotensin II receptor 1 and angiotensin converting enzyme expression, thus leading to a vicious cycle (Keidar et al., 2005; Pitt, 2012). Typically, the effects of the MR in the cardiovascular tissue are very versatile and context-dependent, indicating additional levels of regulation beyond straightforward gene expression.

For instance, in vessels and *in vivo*, MR activation can lead to both vasodilation or vasoconstriction depending on the experimental setup (Schmidt et al., 2003; Uhrenholt et al., 2003; Nagata et al., 2006; Schmidt et al., 2006; Skott et al., 2006). This suggests that MR effects may be regulated cell-type and context-dependent by external micro-milieu factors. In pathological experimental animal models, MR leads to a series of pathophysiological alterations in the cardiovascular system that mimic aging-dependent changes like an increase in blood pressure, vasoconstriction, oxidative stress or inflammation. Furthermore, an inverse correlation between white blood cell telomere length, a biomarker for oxidative stress and inflammation, and aldosterone levels has been found in normotensive men (Benetos et al., 2005). In patients with cardiovascular disease, the telomere length was reduced, suggesting aldosterone promotes cardiovascular diseases.

To investigate the underlying molecular mechanisms, the MR was studied further in isolated vessels, VSMCs and endothelial cells. In rat aorta and in isolated rat VSMCs, MR expression increased with aging and enhanced the expression of pro-inflammatory genes like ICAM, TGFβ and pro-collagen1 via epidermal growth factor receptor (EGFR) signaling (Krug et al., 2010). MR expression was also increased with aging in mesenteric resistance arteries of mice (DuPont et al., 2016). A VSMC specific MR-KO mouse model demonstrated that VSMC-MR leads to increased vascular remodeling after an injury as well as to increased myogenic tone, vasoconstriction and oxidative stress and thereby may promote the rise in blood pressure with aging (McCurley et al., 2012; DuPont et al., 2016). Additionally, the aging-associated increase in vascular fibrosis and stiffness was prevented by the VSMC-specific MR-KO model (Kim et al., 2018).

Healthy endothelial cells play an important anti-inflammatory and antithrombotic role in the vasculature and convey vasorelaxation. Under physiological conditions, the role of the MR seems to be vasoprotective, but it is not as well characterized as in models with cardiovascular risk factors (Nietlispach et al., 2007; Hwang et al., 2016). In these pathophysiological models, MR aggravated cardiovascular diseases and MR antagonists were beneficial in helping to restore normal endothelial and vascular function (Farquharson Colin et al., 2000; Garg et al., 2015; Chen et al., 2016). Again, this suggests, external factors can regulate MR function to confer the switch from physiological to pathophysiological functions. Pathological MR effects include initiation of endothelial dysfunction, inflammation and oxidative stress by activating NADPH oxidase, inhibiting NO activity and reducing the expression of protective glucose-6-phosphate dehydrogenase (Hashikabe et al., 2006; Nagata et al., 2006; Leopold et al., 2007; Iwashima et al., 2008; Fels et al., 2010; Maron et al., 2012). Additionally, increased uncoupling of NO synthase has been described, again leading to an increase in ROS (Taylor et al., 2003). Besides enhancing reactive oxygen species (ROS) formation, MR also augmented the expression of cell adhesion molecules like ICAM and VCAM that support inflammation (Caprio et al., 2008; Deuchar et al., 2011). In an atherosclerosis model, endothelial MR was involved in plaque inflammation, leukocyte-endothelial interaction and vascular inflammation, and a specific endothelial cell MR-KO could prevent these changes (Moss et al., 2019). Furthermore, the expression of pro-inflammatory cytokines was enhanced by MR activation (Jia et al., 2016). On the one hand, in models of vascular injury, MR was reported to be pro-thrombotic while, on the other hand, overexpression of MR in endothelial cells increased protein C expression and thereby attenuated thrombin generation and vascular thrombosis (Bodary et al., 2006; Gromotowicz et al., 2011; Lagrange et al., 2014). Aldosterone also induced changes in the expression of epithelial sodium channels, which led to enhanced sodium transport and changes in mechanical cell properties, which contribute to vascular stiffness and fibrosis (Kusche-Vihrog et al., 2008; Drueppel et al., 2013; DuPont et al., 2014; Jia et al., 2016). Substantial sex-dependent differences were reported (Moss et al., 2019). Taken together, MR has diverse effects in the vasculature, which seem to be highly dependent on cellular context and micro-milieu factors, cell-type, sex and experimental setup. Because detrimental effects of the MR are especially pronounced in aged or pre-injured tissues or in the presence of risk factors, we hypothesize that changes in micro-milieu during aging or diseases may influence MR activity by affecting its signaling through induction of posttranslational modifications.

## Posttranslational Modifications of the Mineralocorticoid Receptor

### Phosphorylation

**FIGURE 2:**
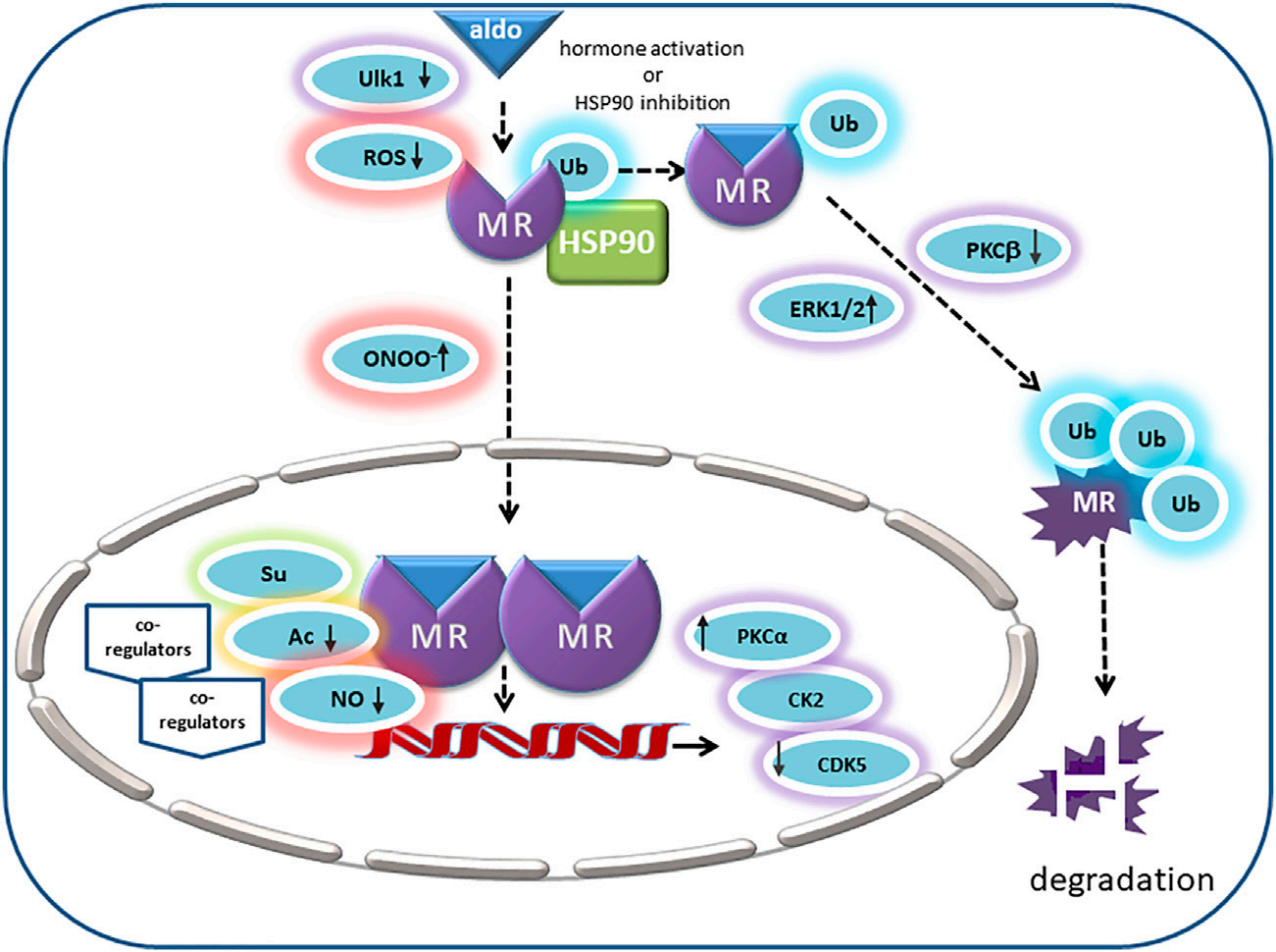
In its unliganded state the MR is monoubiquitinated and located in the cytosol with chaperone molecules like HSP90. Its endogenous ligand aldosterone binds to MR in the cytosol and thereby triggers nuclear translocation. Ligand binding is inhibited by MR oxidation. In intercalated cells, autophagy activating kinase 1 (Ulk1) phosphorylates MR S843 in the LBD, mitigates its affinity for agonists and reducing its transcriptional activity. Peroxynitrite (ONOO-) can cause ligand-independent nuclear translocation and activation of MR. Inhibition of HSP90 or activation by aldosterone causes polyubiquitination of MR and degradation. ERK1/2 and PKC also modulate MR polyubiquitination and degradation. Positive regulators of MR transactivation activity are PKCa and CK2. In the prescence of cytokines, CK2 inhibits GRE signaling but promotes NFKB signaling and transcription of pro-inflammatory cytokines. CDK5 inhibits MR transactivation in neuronal cells. Acetylation (Ac) and SUMOylation (Su) lead to modulation of aldosterone-activated MR transactivation by direct and indirect effects. NO attenuates binding of MR to DNA, consequently reducing the MR’s transcriptional activity (↑ = stimulatory effect; ↓ = inhibitory effect).

Posttranslational modification of steroid receptors allows fine-tuning of receptors stability, ligand-binding, transformation, co-regulator binding, DNA-binding and transactivation (Figure 2). Early studies have shown that the glucocorticoid and estrogen receptor exist as phosphoproteins (Migliaccio et al., 1982; Sanchez et al., 1985; Bodwell et al., 1991). For MR first evidence comes from MR overexpressed in Sf9 cells, where Western blots of precipitated hMR possessed three major protein bands. Pulse-labeling with 32Pi indicated that the recombinant hMR is highly phosphorylated (Alnemri et al., 1991). Galigniana confirmed that native rat kidney MR is a phosphoprotein by showing that incubation with active phosphatase diminished aldosterone binding but accelerated the receptor transformation to a DNA binding form. Endogenous phosphatase could be inhibited by okadaic acid and tautomycin, indicating that it is a serine/threonine phosphatase belonging to the PP1/PP2A subgroup (Galigniana, 1998; Piwien-Pilipuk and Galigniana, 1998). A compact disk (CD)-based microfluidic method for selective detection of phosphopeptides by mass spectrometry identified two phosphorylation sites, Thr 735 and Ser 737, in the ligand-binding domain of the human mineralocorticoid receptor (Hirschberg et al., 2004). Next, in MR-overexpressing COS-7 cells, phospho-peptides were purified and analyzed by LC-MS/MS. This approach revealed sixteen phosphorylation sites of the MR under basal conditions, fourteen of which were located in the NTD, one in the hinge region and one in the LBD (Shibata et al., 2013). Predicted kinases for these sites are proline-directed kinases, glycogen synthase kinase-2, casein kinase II (CK2) and calmodulin kinase II (Shibata et al., 2013).

Of these phosphorylation sites, S843 in the LBD of the MR has been studied in most detail (Shibata et al., 2013). Phosphorylation of MR-S843 resulted in MR inactivation and reduced its ligand binding affinity. It occurred only in intercalated cells of the distal nephron of the kidney and controlled the response to volume depletion and hyperkalemia. In volume depletion, angiotensin II and WNK4 signaling decreased MR-S843 phosphorylation levels, resulting in an increase in apical proton pumps and Cl^−^/HCO_3_^−^ exchangers, which prevented a lumen-negative potential from forming. Consequently, adjacent principal cells promoted an increase in sodium and water reabsorption but no K^+^ secretion. Hyperkalemia, on the other hand, increased phosphorylation of MR-S843 and thereby inactivated aldosterone effects in intercalated cells so that a lumen-negative potential with resulting K^+^ secretion occurred in the renal collecting duct. This mechanism serves as a switch allowing aldosterone to exert distinct effects in different physiological contexts (Shibata et al., 2013). With a high-throughput screening assay and confirmed by *in vitro* and *in vivo* analyses, ULK1 was identified as the principal kinase responsible for the phosphorylation of MR-S843. It was shown to be highly abundant in the intercalated cells of the collecting duct and could be inhibited by angiotensin II via mTOR signaling (Shibata et al., 2018). The group of Alvarez de la Rosa investigated this phosphorylation site with the help of phospho-deficient and -mimetic mutants of mouse MR-839 corresponding to human MR-S843. A phospho-mimetic mutant of hMR-S843 lowered its affinity for agonists, slowed down its ligand-induced translocation and abrogated its transcriptional activity. Co-factor recruitment was impaired as shown for SRC-1 by proximity ligation assay. MR stability and dimerization were not affected (Jimenez-Canino et al., 2016a). A phospho-mimetic MR mutant displayed a dominant-negative effect when forming heterodimers with wild type MR, indicating that phosphorylation of one MR per homodimer is sufficient to impair transcriptional activity so that the overall effect is amplified.

As another functionally relevant kinase, ERK1/2 and its effect on MR signaling were studied in renal epithelial cells. Aldosterone led to a rapid phosphorylation of MR that was detected by a phosphatase-sensitive shift in gel band size and that could be abrogated by an inhibitor of ERK1/2 phosphorylation. There are six conserved predicted ERK1/2 phosphorylation sites in the NTD of the MR and only mutation of all six diminished the shift in band size and abolished the signal detected with an anti-phosphoserine antibody. Functionally, MR phosphorylation led to the disruption of the interaction between MR and tumor susceptibility gene 101 (Tsg101) found under basal conditions. Subsequently, removal of a monoubiquitin, followed by polyubiquitination and degradation of the MR took place (Faresse et al., 2012). Since aldosterone via MR can non-genomically induce ERK1/2 phosphorylation, this may be a negative feedback system involved in limiting for example aldosterone-induced sodium reabsorption (Grossmann et al., 2004; Grossmann et al., 2005; Faresse et al., 2012).

Additionally, there are some indications that different PKC isoforms can affect MR signaling through direct phosphorylation of the receptor. Aldosterone triggered both an early nongenomic and a late genomic increase in sodium transport in rat cortical collecting duct cells dependent on PKCα. The early aldosterone-induced increase in short-circuit current involved rapid phosphorylation of MR on serine and threonine residues, which could be abolished by a PKCα inhibitor. In contrast, the late aldosterone-induced increase in ion transport and gene expression required protein synthesis but could also be inhibited by blocking the PKCα pathway so that a crosstalk between rapid and prolonged MR effects was suggested (Le Moellic et al., 2004). In contrast, stimulation of PKCβ signaling, for example, by high glucose concentrations, led to a reduction in MR ubiquitination and degradation, which caused elevated MR levels and transcriptional activity (Hayashi et al., 2017). For PKCδ, angiotensin II led to MR-PKCδ complex formation in association with increased serine phosphorylation of the MR-NTD in vascular cells. AngII also activated MR transcriptional activity, target gene expression, and SMC proliferation in a PKCδ-dependent manner that may involve phosphorylation of MR-NTD (Lu et al., 2019). Although MR phosphorylation by PKC isoforms has been demonstrated by several groups, it is not always clear if the effects described evolve from a direct MR phosphorylation or indirectly through phosphorylation of co-regulators or a combination of both.

Another potential kinase for MR phosphorylation is the ubiquitously expressed serine/threonine kinase CK2. Inhibition of CK2 reduced the shift in gel band size of MR caused by aldosterone and inhibited MR transcriptional activity under control conditions (Ruhs et al., 2017). Peptide microarrays and site-directed mutagenesis experiments identified the highly conserved S459 as a functionally relevant CK2 phosphorylation site for MR signaling (Ruhs et al., 2017). Consequently, CK2 seems to act as a positive modulator of MR transcriptional activity by phosphorylating the MR. Further investigations unveiled that CK2 facilitates the MR-DNA interaction with subsequent rapid MR degradation. In the presence of pro-inflammatory cytokines, MR and CK2 expression were enhanced, and MR activation by aldosterone augmented CK2-dependent NF-κB signaling and enhanced the expression of pro-inflammatory genes. This may be a mechanism to explain MR over-activation in a pathological micro-milieu.

In neuronal cells, MR has an impact on neuronal viability, synaptic plasticity and emotional changes. There, CDK5 interacted with the LBD of the MR and aldosterone-dependently phosphorylated the MR-NTD residues S128, S250, and Th159, identified by mass spectrometry analysis and mutation experiments. Phosphorylation of MR reduced its transcriptional activity in an MMTV-reporter assay but did not affect nuclear accumulation. On the other hand, aldosterone and CDK5 increased the expression of brain-derived neurotrophic factor in rat cortical neuronal cells (Kino et al., 2010). This suggests that CDK5 through phosphorylation mediates the interaction of the MR with co-regulators and thereby modulates its transcriptional activity without affecting hormone binding or receptor translocation, similar as has been demonstrated for the GR (Kino et al., 2007).

Other kinases for which a regulatory role on MR signaling through phosphorylation has been postulated include PKA and G protein-coupled receptor kinases (GRK). Activation of cAMP-PKA signaling stimulated GRE-containing promoters in a ligand-independent manner and also synergistically after aldosterone activation. Protein kinase-inhibiting peptide (PKI) prevented both cAMP and aldosterone induction, which indicates that a functional cAMP pathway is required for MR transactivation activity. Binding of the MR to a GRE-containing oligonucleotide in a gel shift assay was enhanced by PKA and required the NTD of the MR. However, purified MR NTD protein was not directly phosphorylated by PKA *in vitro* so that PKA seems to act indirectly, probably by relieving the effect of an MR repressor (Massaad et al., 1999). Conversely, MR-S601, predicted to be a phosphorylation site for PKA or for casein kinase I, has been identified as a phosphorylation site by mass spectrometry under basal conditions by another group (Shibata et al., 2013). It is located within the NLS0 of the NTD and consequently affects nuclear translocation. Phospho-mimetic mutants of MR-S601 restricted the MR to the cytosol while phospho-deficient mutants shifted the MR to the nucleus (Walther et al., 2005). *In vivo* implications of these findings have not been studied so far. Last, GRK2 and -5 were also shown to phosphorylate aldosterone receptors. While GRK5 was reported to phosphorylate and thereby inhibit cardiac MR, GRK2 phosphorylated and desensitized the aldosterone membrane receptor GPER. In H9C2 cardiomyocytes and adult rat ventricular myocytes, β_2_-adrenergic receptor activation led to phosphorylation of MR by GRK5, which inhibited the transcriptional MR activity. GRK5 was necessary for the cardioprotective effects of MR antagonists against aldosterone-induced apoptosis and oxidative stress. Conversely, GRK2 seemed to oppose the beneficial effects of aldosterone mediated by GPER signaling (Maning et al., 2020). However, the role of GPER for overall pathophysiological aldosterone effects is still under debate. Supplementary Table S1 summarizes phosphorylation sites, kinases and effects.

### Acetylation

Acetylation is a general epigenetic modification responsible for controlling the activity of various cytosolic and nuclear proteins involved in gene expression like histones, nuclear receptors and chaperones (Kovacs et al., 2005; Murphy et al., 2005; Minucci and Pelicci, 2006). It is a reversible modification depending on the balance between lysine acetyltransferases (KAT) and lysine deacetylases (KDAC) also known as histone acetyltransferases and histone deacetylases (HDAC). Aberrant acetylation of lysine residues has previously been linked to diseases, and inhibitors of HDAC exhibit antifibrotic, anti-inflammatory, anti-hypertrophic, and anti-hypertensive effects (Minucci and Pelicci, 2006; Cardinale et al., 2010; Iyer et al., 2010; Schneider et al., 2013; Seo et al., 2015). Acetylation of the N-terminus of histones is essential to expose promoter DNA to transcription factors and RNA polymerase II for initiating transcription. Acetylation of nuclear receptors controls a wide variety of cellular functions, including nuclear receptor activity, DNA binding affinity, ligand sensitivity, receptor stability, and subcellular distribution (Wang et al., 2011). GR, progesterone receptor, androgen receptor and MR share a KXKK acetylation motif in the hinge region within NLS1 (Faus and Haendler, 2006), suggesting a common regulatory mechanism. GR deacetylation in this region facilitates NF-κB suppression (Ito et al., 2006) and regulates the expression of genes involved in generating circadian rhythm (Nader et al., 2009). For the MR, acetylation at K677 in the NLS1 of the hinge region has been demonstrated. Mutated MR without the lysine residues in the hinge region showed less acetylation and more transcriptional activity (Lee et al., 2013). Overexpression of CBP and p300 in the presence of aldosterone led to increased acetylation of MR in the nucleus, suggesting they are the responsible lysine acetyltransferases (Pascual-Le Tallec and Lombes, 2005; Seo et al., 2015). Although aldosterone treatment enhanced the nuclear availability of the MR, it did not affect the subcellular localization of p300 or CBP (Seo et al., 2015). Increased acetylation led to decreased MR transcriptional activity with reduced expression of MR target genes and attenuated MR and Pol II recruitment to specific hormone response elements. No effect on MR expression or translocation was found (Lee et al., 2013; Kang et al., 2015; Lee et al., 2015; Seo et al., 2015; Seok et al., 2016). Besides having lysine acetyltransferase activity, CBP and p300 can also function as transcriptional adaptors and thereby MR co-activators to enhance MR activity (Fuse et al., 2000; Peter et al., 2017). Therefore, CBP and p300 context-dependently seem to act as co-regulators of the MR. Only inhibition of HDACI but not of HDACII activity increased MR acetylation and attenuated MR target gene expression (Lee et al., 2015). Of the lysine deacetylases in group I, HDAC3 was shown to interact with MR and to reduce its transcriptional activity. Accordingly, HDAC3 knockdown increased MR acetylation and decreased MR target gene expression by inhibiting the MR interaction with hormone-responsive-elements and RNA polymerase (Lee et al., 2013). Furthermore, HDAC4 was identified as a necessary scaffolding protein between MR and HDAC3. Non-genomic effect of MR via PKA and PP1/2 induced nuclear translocation of HDAC4 to facilitate the interaction between MR and HDAC3. Lower levels of HDAC4 expression led to a decreased interaction between MR and HDAC3, promoting increased MR acetylation and decreased transcriptional activity (Lee et al., 2015).

Accordingly, the HDACI inhibitor valproic acid (VPA) increased MR acetylation and decreased MR transcriptional activity in reporter gene experiments. This corresponded to reduced MR target gene expression as well as reduced pol II recruitment to the respective promoters. Pathophysiological relevance of MR acetylation was also demonstrated with HDAC inhibitors. In mCCD cells, aldosterone augmented ENaC-induced Na^+^ absorption and increased SGK1 activity and abundance. HDAC inhibition by trichostatin A could inhibit aldosterone/MR dependent regulation of SGK1/ENAC signaling leading to sodium reabsorption. Insulin-dependent activation of SGK/ENAC signaling was not modified. Consequently, the SGK1-ENAC pathway was not affected by pan HDAC inhibition, but the control of MR over this pathway was perturbed, which again supports that MR acetylation inhibits MR activity (Mansley et al., 2019). Furthermore, HDAC inhibition attenuated pathophysiological MR effects like hypertension, hypertrophy, inflammation and fibrosis *in vivo* in animal models of spontaneous hypertension and hyperaldosteronism (Cardinale et al., 2010; Iyer et al., 2010; Lee et al., 2013; Kang et al., 2015; Seok et al., 2016). Although treatment with VPA not only enhanced MR acetylation but also increased histone-3-acetylation (H3Ac) and trimethylation (H3K4me3) in the promoter regions of MR target genes, expression of MR target genes was decreased, and the development of hypertension in deoxycorticosterone acetate-induced hypertensive rats and spontaneously hypertensive rats (SHR) was prevented (Seok et al., 2016). These results suggest that HDAC inhibition attenuates the development of hypertension in SHR through acetylation of MR and independent of histone effects. Treatment with VPA also histologically attenuated cardiac hypertrophy and fibrosis through acetylation of MR in spontaneously hypertensive rats (Kang et al., 2015). An indirect effect of acetylation on MR signaling was also reported for its chaperone HSP90. Increasing acetylation of residue K295 of HSP90 by inhibiting HDAC6 weakened HSP90′s interaction with the MR and supported MR nuclear shuttling. However, HSP90-K295 acetylation did not alter MR expression or transactivation activity (Jimenez-Canino et al., 2016b).

### Ubiquitination

Ubiquitin is covalently conjugated to lysine residues of substrate proteins through an ATP-dependent reaction that involves successive action of ubiquitin-activating enzyme E1, ubiquitin-conjugating enzyme E2, and ubiquitin ligases E3. Either monoubiquitination with a single ubiquitin molecule or polyubiquitination with ubiquitin chains is possible. Ubiquitin itself can be ubiquitinated further or ubiquitin-like molecules like SUMO or NEDD8 can modify it. Additional posttranslational modification through acetylation or phosphorylation is possible, and each modification may lead to an altered signaling outcome. For example, covalent attachment of ubiquitin polypeptide often promotes protein turnover by targeting the protein to the proteasome for degradation. The ubiquitin-proteasome pathway regulates the turnover of many nuclear hormone receptors and decreases their ligand-mediated transcriptional activity (Wallace and Cildlowski, 2001; Yokota et al., 2004). For the GR, proteasome activity is required for rapid GR exchange at promoters (Stavreva et al., 2004). Treatment with proteasome inhibitor enhanced GR activity and increased *in vivo* DNA occupancy time, indicating that ubiquitination is involved in regulating GR stability (Deroo et al., 2002; Stavreva et al., 2004). Furthermore, ubiquitination of GR at Lys419 was shown to stimulate GR nuclear export and subsequent degradation (Kino et al., 2004; Wallace et al., 2010). Yokota et al. first showed that treatment with aldosterone leads to reduced MR expression and targeting of the MR to the proteasome (Yokota et al., 2004). Accordingly, proteasomal inhibition prevented ligand-dependent degradation of MR and thereby enhanced its transcriptional activity. However, mutation of the lysines of two PEST motifs predicted as candidate ubiquitination sites, K715 and K367, failed to prevent degradation and therefore other residues or indirect effects seem to be responsible (Yokota et al., 2004). Later it was demonstrated that under basal conditions, MR is monoubiquitinated, and this state is stabilized by Tsg101 (Burgdorf et al., 2004; Ismaili et al., 2005; La Rosa et al., 2011). In the cytosol, polyubiquitination of unstimulated MR is prevented by its interaction with HSP90. Inhibition of HSP90 promoted polyubiquitination through interaction with the ubiquitin-protein ligase CHIP, which initiated proteasomal degradation of unliganded MR and reduced MR protein levels as well as aldosterone-dependent transcription and sodium transport (Faresse et al., 2010). In renal M1 cells, USP2-45, a ubiquitin-specific protease, was responsible for removal of monoubiquitin. USP2-45 down-regulation or USP-2 deficiency led to increased MR protein expression *in vitro* and *in vivo*. *In vivo* this increase in MR protein did not lead to physiological disturbances (Faresse et al., 2013). Aldosterone also led to disruption of MR/Tsg100 association and removal of monoubiquitin followed by polyubiquitination and MR degradation. Nevertheless, polyubiquitination and proteasome activity seemed essential for nuclear mobility and MR transcriptional activity (Tirard et al., 2007). The exact target lysines and ligases are not fully clear. Phosphorylation of MR via ERK1/2, which can be induced by non-genomic MR effects, also promoted monoubiquitin removal and MR degradation (Yokota et al., 2004; Tirard et al., 2007; Faresse et al., 2012). Conversely, aldosterone-mediated MR degradation could be prevented by ERK1/2 inhibition or mutation of target serines (Faresse et al., 2012), indicating a possible negative feedback loop. On the other hand, *in vitro* and *in vivo* experiments showed that ERK activation in the kidney through EGFR activation counteracted MR ubiquitination and increased MR protein expression and transcriptional activity. EGFR inhibition lowered systolic blood pressure and MR activity in DOCA/salt-treated mice (Mitsuishi et al., 2018). Another cytoplasmic binding partner of MR that promotes receptor accumulation and aldosterone-induced sodium uptake in renal cells due to less proteasomal degradation of MR is protein phosphatase 1 alpha (PP1α). To achieve this, PP1α dephosphorylates and thereby inhibits the ubiquitin ligase Mdm2 and thereby prevents its interaction with MR (Nagarajan et al., 2017). As mentioned previously, activation of PKCβ by high glucose concentrations also increased MR expression and transcriptional activity by increasing phosphorylation and decreasing ubiquitination of MR. Accordingly, in kidneys of db/db mice, a type 2 diabetes mouse model with PKCβ activation, levels of MR and Sgk-1 were elevated and could be reversed by PKC inhibition. PKC inhibition also lowered systolic blood pressure (Hayashi et al., 2017).

### Sumoylation

Sumoylation involves covalent attachment of small proteins called small ubiquitin modifiers (SUMO), which possess four isoforms SUMO1-4 in humans. SUMOs are preferentially attached to lysine in the consensus sequence ΨKD/E and share a similar structure with ubiquitin. As with ubiquitination, attachment requires three enzymes E1-E3. The MR contains five consensus SUMO-1 binding sites, four in the AF1 domain of the NTD and one in the LBD. PIAS1 and some of its family members can function as E3 ligases and are able to interact with the MR. They can directly sumoylate the MR as demonstrated by multiple shifted bands in Western blot analyses, which are missing after mutating respective amino acids. Overexpression of PIAS-1, which led to increased MR sumoylation, had no effect on MR expression or subcellular localization but reduced its transcriptional activity upon ligand binding. The inhibition was equally strong after deleting the first 453 amino acids of the NTD but was not present for GR and dexamethasone (Pascual-Le Tallec et al., 2003). Mutation of all four NTD-SUMO-1 attachment sites abolished MR sumoylation and enhanced transcriptional activity of aldosterone-stimulated MR at compound GRE elements while nuclear mobility was decreased. However, transcriptional activity at MMTV promoter elements was unchanged after aldosterone treatment (Tirard et al., 2007). Because SUMO-1 overexpression also led to inhibition of the activity of sumoylation-deficient MR at a compound GRE element, this suggests that additional MR cofactors also get sumoylated and modulate transcriptional MR activity (Pascual-Le Tallec et al., 2003; Tirard et al., 2007). The NTD of the MR not only interacted with PIAS but also with SUMO-1 and Ubc9, a SUMO-E2 conjugating enzyme (Pascual-Le Tallec et al., 2003; Tirard et al., 2007). Overexpression of Ubc9 increased MR transactivation at MRE, ENaC, or MMTV promoter elements in a hormone-sensitive manner, and knockdown reduced expression of MR target genes. However, Ubc9 showed similar effects when applying a sumoylation-inactive Ubc9 mutant or an MR with four mutated sumoylation motifs, indicating that Ubc9 acts as a transcriptional co-activator beyond its SUMO E2-conjugating function. Accordingly, MR, Ubc9, and the steroid receptor co-activator SRC-1 formed a complex and were recruited in an aldosterone dependent manner to endogenous ENaC gene promoters, where they synergistically enhanced MR transactivation activity (Yokota et al., 2007). Another factor interacting with Ubc9, SUMO1 and MR is FAF1, an MR co-repressor, which contains two SUMO interacting motifs with which it binds to sumoylated MR and thereby represses its aldosterone-activated transactivation. Silencing FAF1 expression augmented MR target gene expression. Mechanistically, FAF1-mediated MR transrepression involved inhibition of MR N/C terminal interactions and promotion of MR polyubiquitination and degradation (Wang et al., 2019). Additionally, an indirect effect on MR signaling was demonstrated by sumoylation of 11β-HSD2 at lysine 266. Despite having no effect on protein stability or subcellular localization and only a slight effect on enzyme kinetics, sumoylation decrease translocation of MR upon cortisol treatment without affecting transcriptional activity (Jimenez-Canino et al., 2017). Overall, sumoylation of MR and of interaction partners seems to be important for regulating the interaction of MR with co-regulators again in a context- and promoter-dependent manner. For the GR it is also known that it contains two sumoylation sites in the NTD and one in the LBD. Sumoylation in the NTD leads to a similar context-dependent inhibition of receptor transcriptional activity with no clear effect at MMTV promoters. The third sumoylation site is involved in stimulating GR activity under conditions of cellular stress. Furthermore, FKBP51 was sumoylated under these conditions and supported GR-FKBP51 complex formation and interaction with HSP90, which promoted cytosolic, inactive GR. A similar interaction seems likely for the MR. Other steroid receptors show a similar sumoylation of the NTD, which modulates their respective transcriptional activity (Poukka et al., 2000). The data suggest that the overall transcriptional activity of MR can be modulated by direct sumoylation as well as the sumoylation of MR-interacting proteins (Tirard et al., 2007).

### Oxidation

During aging, oxidized proteins accumulate in tissues because of increased ROS generation and a reduced antioxidative capacity (Stadtman, 2006). Overall, the expression of the MR in target tissues is low, its HSP90 heterocomplex easily dissociates, and it is highly susceptible to proteolysis and oxidation (Galigniana and Piwien-Pilipuk, 1999a). Oxidation of MR has been shown to prevent aldosterone and DNA binding of the receptor and to attenuate biological MR effects, all of which can be reversed or attenuated by reducing agents (Galigniana, 1996; Piwien-Pilipuk and Galigniana, 2000; Piwien-Pilipuk et al., 2002; Ruhs et al., 2012). Reduced aldosterone- and DNA-binding of MR caused by H_2_O_2_ was enhanced by iron and inhibited by sulfhydryl reducing reagents, suggesting that disulfide bonds of cysteines are responsible (Galigniana, 1996). Treatments with metals *in vitro* and *in vivo* in adrenalectomized rats inhibited both steroid-binding capacity and aldosterone-dependent sodium-retaining properties (Galigniana and Piwien-Pilipuk, 1999a). As a mechanism leading to loss of steroid binding capacity and MR function, receptor oxidation at essential cysteines was identified because a decreased number of reactive thiols was found on immunopurified receptors after *in vivo* glutathione depletion (Piwien-Pilipuk and Galigniana, 2000). Furthermore, treatment of cytosolic MR of Sf9 cells with a cysteine modifying agent inhibited the binding of hMR to [^3^H]aldosterone. Agonist-bound MR was resistant to cysteine modifying agents while free hMR and antagonist-bound hMR was sensitive. Therefore, sulfhydryl groups are involved in binding of ligands to the MR, and their accessibility varies depending on the ligand-status of the MR and the receptor conformation (Souque et al., 1996). Another mechanism leading to reduced ligand-binding and MR function in a glutathione depletion model was enhanced MR and EF-2 carbonylation with inhibited protein translation and MR effects (Piwien-Pilipuk and Galigniana, 2000; Piwien-Pilipuk et al., 2002). Association with chaperone complex was not affected by MR oxidation (Piwien-Pilipuk et al., 2002). Overall, depletion of glutathione in kidney cells was shown to mimic the cumulative effect of aging (Piwien-Pilipuk et al., 2002).

Not only oxidative stress but also nitrosative stress occurs during aging and affects MR signaling. For the GR it has been shown that NO can react with SH groups to form S-nitrosothiols, which inhibit binding of GR to ligand without affecting MR-HSP90 interaction (Galigniana et al., 1999b). For the MR, NO led to a reduction of MR and GR transactivation activity upon corticosteroid treatment (Ruhs et al., 2012). In contrast, peroxynitrite administration induced ligand-independent MR transactivation, whereas genomic GR activity remained unchanged. This effect was mediated by ligand-independent nuclear translocation of peroxynitrite-treated MR (Ruhs et al., 2012). On the other hand, aldosterone via the MR can also activate NADPH oxidases in vascular cells and thereby increase ROS levels, leading to posttranslational modification of other proteins (Hashikabe et al., 2006). For instance, posttranslational modification of Cys-122 of the guanylyl cyclase can inhibit GC activity by modulating NO sensing. A C122A-GC-mutant exposed to aldosterone or hydrogen peroxide did not show increased cGMP levels like WT (Maron et al., 2009). In hPAEC, endothelin-1 increased aldosterone levels via upregulation of aldosterone synthase. Aldosterone then increases ROS production, which oxidatively modified cysteinyl thiols in the endothelial NO synthase-activating region of the endothelin-B-receptor, thereby reducing endothelin-1-mediated endothelial NO synthase activity. An MR antagonist restored Endothelin-B-receptor-mediated NO production. *In vivo* in two rat pulmonary arterial hypertension models, MR antagonists prevented or reversed pulmonary vascular remodeling. There elevated endothelin-1 levels were associated with elevated aldosterone levels in plasma and decreased lung NO metabolites (Maron et al., 2012).

## Discussion

**FIGURE 3:**
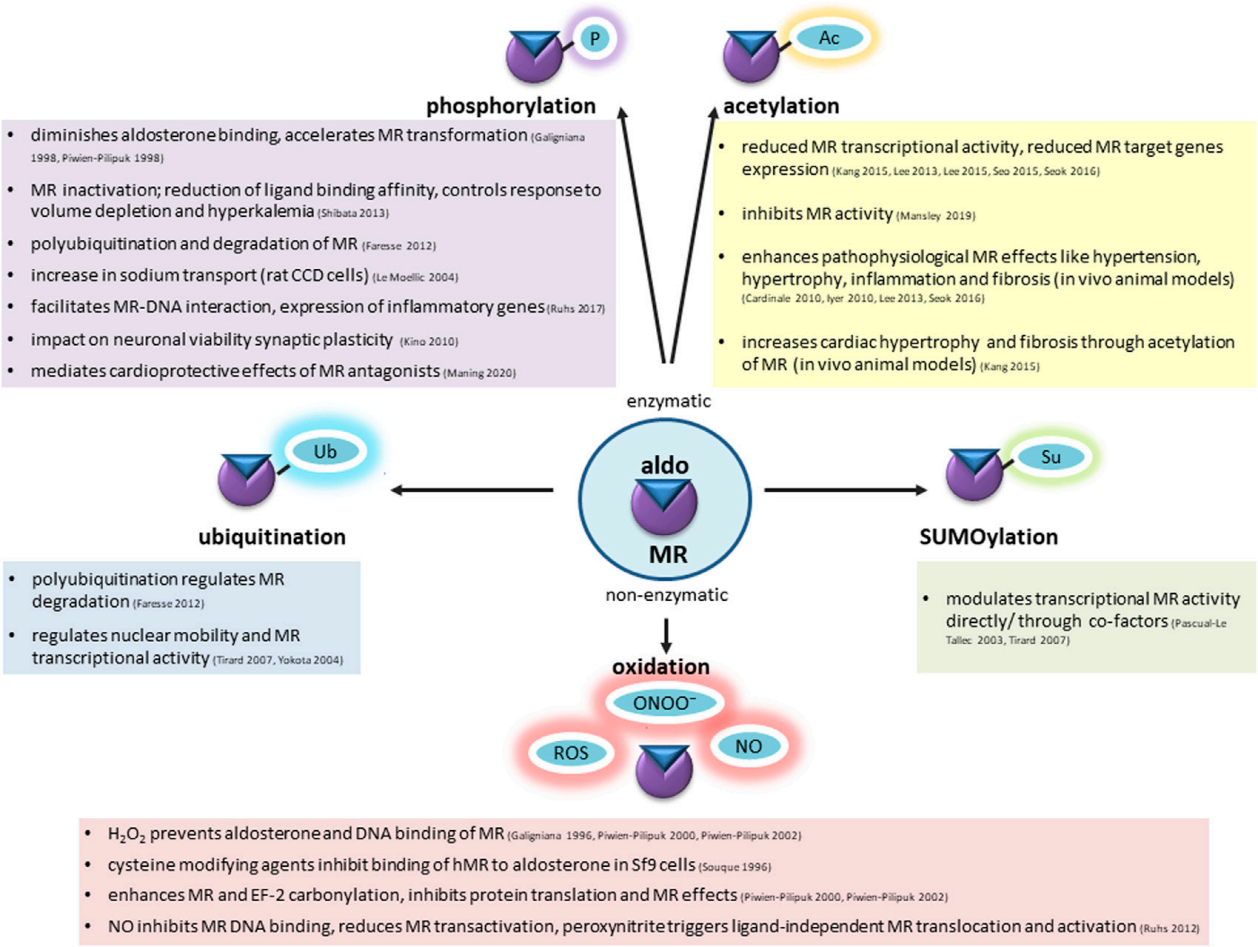
Mineralocorticoid receptor undergoes enzymatic and non-enzymatic posttranslational modifications, affecting the basal condition of the receptor, ligand binding, shuttling to the nucleus, DNA interaction and transactivation activity. Among enzymatic modifications phosphorylation (P), acetylation (Ac), ubiquitination (Ub), SUMOylation (Su) has been well-described. Increased ROS and RNS production during aging causes non-enzymatic alterations. Possible consequences are summarized in this figure.

Besides exerting classical epithelial effects on volume-and electrolyte homeostasis, the MR has a large impact on cardiovascular pathophysiology with cell-type-specific and context-dependent functions. While ensuring homeostasis in young and healthy tissues, the MR tends to aggravate pathological changes in aged or pre-injured tissues exposed to cardiovascular risk factors. It is unclear what causes the switch from physiological to pathological functions, but one possibility is posttranslational modification induced by a pathological micro-milieu. Signaling of the MR resembles that of its steroid receptor relatives and involves cytosolic association to chaperones, binding of ligand, nuclear translocation and function as a transcription factor. For the MR, many variants are known and have contributed to the understanding of MR structure and function. Loss of function mutations, which in the extreme cause pseudohypoaldosteronism type 1, and gain-of function-mutations, which give rise to inherited forms of hypertension, usually lead to structural alterations of the MR that affect its fundamental functions like binding of ligand or DNA (Zennaro and Fernandes-Rosa, 2017). Over 380 rare coding variants have been described in the major MR transcript in the Exome Aggregation Consortium (ExAC) (Lek et al., 2016). These data indicate that MR is intolerant for missense and nonsense mutations, suggesting that rare variants and common single nucleotide polymorphisms may have functional consequences in the general population and modulate disease susceptibility. Much evidence indicates that MR can be posttranslationally modified, affecting MR signaling and function. The possible impact of MR variants for its posttranslational modifications has not been assessed. Modifications described include enzymatic and non-enzymatic modifications, including phosphorylation (p), acetylation (ac), sumoylation (su), ubiquitination (ub) and oxidation (Figure 3). A direct modification of the MR was proposed for each one, although the exact molecular mechanisms, modifying enzymes and how PTMs are regulated during physiological and pathological situations is not clear. Especially, the *in vivo* relevance of posttranslational modifications for MR function needs further exploration for understanding the mechanisms of cardiovascular diseases and healthy aging.
