# Supplementary material for: Posttranslational Modifications of the Mineralocorticoid Receptor and Cardiovascular Aging
Source: Front Mol Biosci. 2021 May 28;8:667990. doi: 10.3389/fmolb.2021.667990 (PMC8193679; doi:10.3389/fmolb.2021.667990)
Supplement: Supplementary file 1 [file DataSheet1.PDF]

Table 1

location		kinase	methods	reference	effects
**NTD**	S8	casein kinase II	M/S predicted	(Shibata et al., 2013)	?
	S128<br>S250*<br>T159	CDK5	M/S muations	(Kino et al., 2010)	regulates transcriptional activity
	S196<br>S227<br>S238<br>S287<br>S361*	ERK1/2	mutations antibody	(Faresse et al., 2012)	regulates MR transactivation and stability
	S129<br>S183<br>S250*<br>S259<br>S274<br>S283<br>S299<br>S361*	proline-directed kinases	M/S predicted	(Shibata et al., 2013)	?
	S311<br>S424	??	M/S predicted	(Shibata et al., 2013)	?
	S255<br>S262	glykogen synthase kinase3	M/S predicted	(Shibata et al., 2013)	?
	S543	CAMKII	M/S predicted	(Shibata et al., 2013)	?
	S459	CK2	peptide array mutation	(Ruhs et al., 2017)	influences MR-DNA interaction
	S601	PKA or casein kinase I	mutation	(Walther et al., 2005)	affects intracellular localization of MR
	??	PKCδ	antibody	(Lu et al., 2019)	affects MR transactivation
**hinge**	S703	proline-directed kinase	M/S predicted	(Shibata et al., 2013)	?
**LBD**	T735<br>S737		M/S	(Hirschberg et al., 2004)	?
	??	PKCa	antibodies	(Le Moellic et al., 2004)	affects MR transactivation
	S843	ULK1	M/S Mutation S843-P-antibody	(Shibata et al., 2013)	affects ligand binding
